# Recycling and Processing of Waste Materials

**DOI:** 10.3390/ma16020508

**Published:** 2023-01-05

**Authors:** Paulina Wiśniewska, Aleksander Hejna, Mohammad Reza Saeb

**Affiliations:** 1 Department of Polymer Technology, Faculty of Chemistry, Gdańsk University of Technology, Gabriela Narutowicza 11/12, 80-233 Gdańsk, Poland; 2 Advanced Materials Center, Gdańsk University of Technology, 80-233 Gdańsk, Poland; 3 Institute of Materials Technology, Poznań University of Technology, Piotrowo 3, 60-965 Poznań, Poland

Just a few days ago, the world population, as expected, surpassed 8 billion. The message that could be delivered to the nations is reflecting several consequences and concerns, such as the lack of natural resources, health issues, water shortage, and food scarcity, which highlights some real penalties. Not surprisingly perhaps, the myopic attitude of the majority of developing and even developed countries to environmental issues should deepen global concerns in terms of carbon emission, soil erosion, droughts, floods, etc. The feeling of apprehension around the huge amounts of waste produced worldwide should cause alarm. Disciplined monitoring, collecting, classifying, separating, and processing of waste materials via accelerated and reliable waste management strategies seems necessary. Policymakers and researchers are, in a parallel manner, endeavoring to revisit the milestone of waste management by reassessing the current protocols, along with implementing and updating regional and global legislations. Nevertheless, the pathways that have been proposed so far for closing the huge gaps between theoretical and practical guidelines seem to be far beyond the reach of technologists. Thus, actors in waste management should play a crucial role by anticipating the risks and practical solutions rather than merely focusing on science.

From a very general view, waste materials can be classified into plastics, paper, metals, glasses, and organics (Figure 1). Aside from exaggeration and compliments, achieving such an elementary level of waste management still faces serious problems, even in developed countries. Therefore, classifying and separating waste materials as the first step in the sustainable development of waste is pertinent to organizing training protocols in a dynamic manner via an evolutionary amalgamation of multidisciplinary socio-cultural programming. Figure 1 also gives a fast view of waste-processing strategies, mainly including disposal (known as the most generic approach), recovery and recycling (known as the best and most sustainable approach) methods, ended in an ideal green factory with zero emissions at the end of sustainability cycle—this can be the ultimate goal of waste materials management cycle for guaranteed environmental protection. Therefore, circularity from one side dictates organization and processing of waste, while the economy on the other side demands development of value-added products from waste [1]. Materials circularity, circular economy, and life-cycle assessment are some fundamental concepts, which are born accordingly from environmental protection and economy as parents [2]. Both qualitative and quantitative analyses are today centered on the assessment of performance of the recycled materials and recycling techniques.

In line with ever-increasing demands for processing and recycling waste materials [3], this Special Issue, entitled “Recycling and Processing of Waste Materials” has been proposed by the journal of *Materials* from the MDPI family. We, Paulina Wiśniewska, Aleksander Hejna, and Mohammad Reza Saeb, are all dynamically working and practically pursuing recycling and management of waste materials, particularly polymer wastes. “Recycling of waste materials”, “recycling of polymers”, “processing of biowastes”, “conversion of wastes to value-added products”, “upcycling of materials”, “circularity of waste materials”, “techniques of waste materials management”, “additive manufacturing (3D printing) of recycled polymers”, “developing composites and nanocomposites from waste materials”, “sustainability features of waste materials”, “waste processing and circular economy”, and “challenging aspects of the processing of waste materials” are the main but not limited to the topics this Special Issue covers. Therefore, we encourage colleagues from academia and industry alike to consider the aforementioned topics for contribution to this Special Issue. Of note, the possible ways of separation, treatment, processing, and modification of waste materials are centered on our interest. The need for engagement from downstream users to ensure the persistence of practical recommendations besides science-based developments would lead to supportable research in the field. We particularly invite our colleagues from the industry zone who may contribute to this Special Issue to impart their advice on troubleshooting strategies as well.

## Figures and Tables

**Figure 1:**
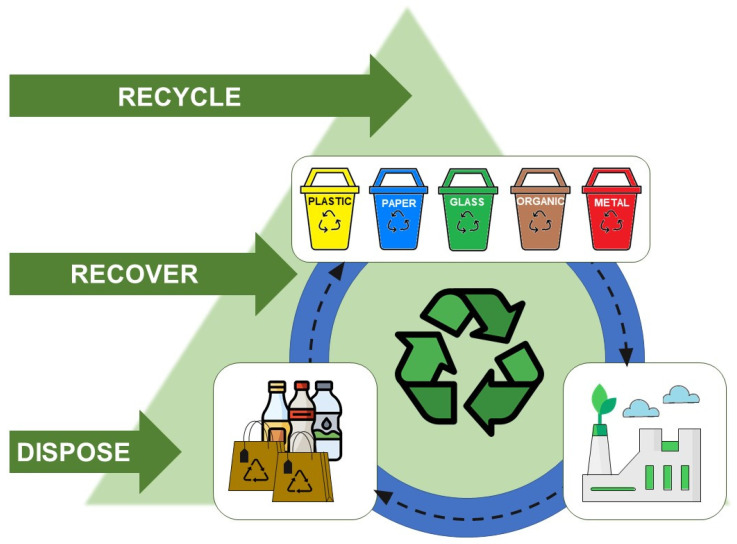
A very general view of waste material sources and waste management strategies, together with waste-processing cycle. By means of this illustrative pattern we emphasize the superiority of recycling strategy rather than disposal and recovery as other solutions provided to waste materials. Indeed, energy management have to be considered in realization of ‘green factory’ horizon shown in this scheme in addition to materials management.
